# ATP11A Promotes Epithelial-mesenchymal Transition in Gastric Cancer Cells via the Hippo Pathway

**DOI:** 10.7150/jca.97895

**Published:** 2024-08-19

**Authors:** Zhihua Wang, Mingmiao Xue, Junqiang Liu, Han Jiang, Feifan Li, Min Xu, Huizhi Wang

**Affiliations:** 1 Department of Gastroenterology, Affiliated Hospital of Jiangsu University, Jiangsu University, 438 Jiefang Road, Zhenjiang 212001, China.; 2 Department of Endocrinology, Affiliated Hospital of Jiangsu University, Jiangsu University, 438 Jiefang Road, Zhenjiang 212001, China.

**Keywords:** ATP11A, EMT, Hippo, gastric cancer.

## Abstract

**Background:** ATP11A, a P-type ATPase, functions as flippases at the plasma membrane to maintain cellular function and vitality in several cancers. However, the role of ATP11A in gastric cancer remains unknown. This study aimed to identify ATP11A related to the biological behavior of gastric cancer, and elucidate the underlying mechanism.

**Methods:** The Cancer Genome Atlas (TCGA) and Genotype-Tissue Expression (GTEx) databases were used to analyze the expression and prognosis of ATP11A. The biofunctions of ATP11A were explored through Gene Ontology (GO), Kyoto Encyclopedia of Genes and Genomes (KEGG) and Gene Set Enrichment Analysis (GSEA). The expression of ATP11A were validated by immunohistochemistry (IHC), qRT-PCR and Western blotting. Transwell, wound healing, CCK8 and colony-formation were to detected the migration, invasion and proliferation of gastric cancer cells. The epithelial-mesenchymal transition (EMT) and Hippo pathway markers were examined by Western blotting.

**Results:** The expression of ATP11A was higher in gastric cancer tissues than in normal tissues, and high ATP11A levels were related to worse prognosis of gastric cancer patients. Additionally, we proved that ATP11A promoted the migration, invasion and proliferation in gastric cancer cells. Furthermore, ATP11A was found to promote EMT by devitalizing the Hippo pathway.

**Conclusion:** ATP11A promoted migration, invasion, proliferation and EMT via Hippo signaling devitalization in gastric cancer cells.

## Introduction

Gastric cancer, a heterogeneous disease characterized by high morbidity and mortality, is one of the most common causes of cancer death globally 1. Although its incidence is declining, it is still an important global healthcare problem due to the lack of effective early detection biomarkers and easy metastasis and recurrence 2, 3. Therefore, clarifying the potential molecular mechanism of gastric cancer progression may provide a new strategy for the diagnosis and treatment of gastric cancer.

Epithelial-mesenchymal transition (EMT) is an important step in tumor progression, metastasis and invasion 4. During the process of EMT, cancer cells gain more aggressive properties and exhibit decreased cell-cell adhesion and increased motility through the loss of cell polarity and epithelial markers expression and the acquisition of mesenchymal markers expression 5-7. For gastric cancer, EMT plays a vital role in metastasis and recurrence 8, 9. For instance, HOXA10 and CCT5 were identified to promote gastric cancer metastasis by inducing EMT 10, 11. Therefore, it is necessary to understand the molecular mechanism of EMT to find a new diagnosis and treatment of gastric cancer.

ATPase Phospholipid Transport 11A (ATP11A), belongs to class VI of the P4-flippase family, is a catalytic component of the P4-ATPase inverting enzyme complex in mammals 12, 13. ATP11A maintains membrane lipid asymmetry for cell biological activities by catalyzing the hydrolysis of ATP and translocating phosphatidylserine (PS) from the outer leaf to the inner leaf of the plasma membrane 14, 15. ATP11A has been shown to be associated with a variety of diseases, including tumors. At present, ATP11A has been studied in several cancer types, including prostate cancer 16, colon cancer 17 and pancreatic cancer 18. However, the role of ATP11A in gastric cancer remains unclear. Hence, we aimed to explore the association between ATP11A and migration, invasion, proliferation and EMT in gastric cancer cells.

In this study, we focused on the role of ATP11A in gastric cancer for the first time. Firstly, we found that ATP11A was abnormally expressed in gastric cancer tissues and cells, and was associated with poor prognosis. Subsequently, we demonstrated that ATP11A promoted the migration, invasion, proliferation and EMT of gastric cancer cells. Mechanistically, ATP11A promoted EMT by Hippo pathway in gastric cancer cells. Our study suggested that ATP11A might be a candidate target for the diagnosis and prognosis of gastric cancer.

## Materials and Methods

### Gene expression data download and analysis

A comprehensive investigation into gene expression profiles across diverse tumor types was undertaken, encompassing 33 distinct malignancies. RNAseq data from the project STAR, derived from The Cancer Genome Atlas (TCGA) database (https://portal.gdc.cancer.gov, accessed on Agu 12, 2023), were procured in per million format. Simultaneously, information pertaining to healthy tissues and cells was sourced from Genotype-Tissue Expression (GTEx) database. The analytical framework involved R software version 4.2.1, leveraging R packages ggplot2 (version 3.3.6), car (version 3.1.0), and stats (version 4.2.1). Statistical analyses, including the Wilcoxon rank-sum test, were conducted to compare data between two groups, and a significance threshold of *P*<0.05 was applied.

### Clinical significance of ATP11A

To ascertain the clinical relevance of ATP11A in gastric cancer, a diverse array of methodologies was employed. Diagnostic Receiver Operating Characteristic (ROC) curves, risk score analyses, calibration and nomogram analysis were executed. ROC analysis was conducted using the pROC package (version 1.18.0), omitting data without clinical information. Cox regression analysis, calibration assessments, and nomogram construction were facilitated by the survival package and rms package (version 6.3‑0). Visualization of risk scores was achieved using the ggplot2 package.

### Co‑expression gene analysis of ATP11A and functional enrichment

The TCGA database was harnessed to extract data on corresponding molecules, which were dichotomized into high and low expression groups (50% vs. 50%). Utilizing the DESeq2 package (1.36.0), differentially expressed genes (DEGs) were computed. The ggplot2 package facilitated the creation of volcano plots and heatmaps, displaying the top 5 positively and negatively correlated genes. Function enrichment analyses, incorporating Kyoto Encyclopedia of Genes and Genomes (KEGG), Gene Ontology (GO), and Gene Set Enrichment Analysis (GSEA), were executed with the clusterProfiler package (version 4.4.4). Enrichment results were visualized using the ggplot2 and GOplot packages.

### Pathological Sample Collection

Between Jun 2023 and Oct 2023, 3 samples of gastric cancer tissues and their matched paracancerous tissues were collected in the Affiliated Hospital of Jiangsu University. This study was approved by the medical ethics committees of Affiliated Hospital of Jiangsu University and implemented in accordance with the Declaration of Helsinki. Patient consent was waived following approval by the ethics committee and no harm to patients was caused.

### Immunohistochemistry (IHC)

The adjacent tissues and tumor tissues were fixed with 10 % formalin, paraffin-embedded, sliced into 4~6μm sections and placed on slides. After dewaxing, hydration and microwave antigen repair, the slides were incubated with 1:100 diluted ATP11A (GeneTex, GTX85170) antibody at 4°C overnight. The secondary antibody was incubated at room temperature for 30 min, stained with DAB substrate and re-stained with hematoxylin. The results can be analyzed by grey density analysis method. Gray density analysis was performed by using Image J software under the same conditions in the same area of tissue sections from different groups and different animals, and then statistical analysis was performed.

### Cell culture

The gastric cancer cell lines SGC-7901, BGC-823, MGC-803 and HGC-27 were maintained by the Institute of Medical Science, Jiangsu University (Zhenjiang, Jiangsu, China). All cells were tested and authenticated by short tandem repeat analysis. Cells were maintained in a humidified incubator at 37 ˚C supplied with 5% CO_2_ and were cultured in DMEM (Meilunbio, Dalian, China) supplemented with 10% fetal bovine serum and 100 mg/ml penicillin (both from Beyotime Institute of Biotechnology).

### Western blotting

Cultured cells were washed with cold PBS and treated with RIPA lysis buffer at 4 °C for 10 min, followed by heating at 100 °C for 10 min, centrifugation at 12 000×g at 4 °C for 10 min. Each lane was loaded with approximately 25 μg of protein, separated by 10% SDS-PAGE, and transferred to PVDF membranes. Membranes were blocked with 5% BSA for 1 h at room temperature, followed by incubation with primary antibodies overnight at 4 °C and with secondary antibody for 1 h at room temperature. An appropriate amount of ECL was uniformly dropped onto the membrane, and the membrane surface was uniformly covered with chromogenic solution. The images were photographed and analyzed using chemiluminescence imaging analysis software. The antibodies were: rabbit anti-ATP11A (GeneTex, GTX85170), rabbit anti-β-Tubulin (Abcam, CAT 21058), rabbit anti-Flag (ABclonal, CAT AE063), rabbit anti-MMP2 (ImmunoWay, CAT YT2798), rabbit anti-MMP9 (ImmunoWay, CAT YT1892), rabbit anti-Snail (Cell Signaling, CAT 3879), rabbit anti-α-SMA (Cell Signaling, CAT 68463), rabbit anti-Vimentin (Cell Signaling, CAT 5741), rabbit anti-N-cadherin (Cell Signaling, CAT 13116), rabbit anti-β-catenin (Cell Signaling, CAT 8480), rabbit anti-E-cadherin (Cell Signaling, CAT 3195), Hippo Signaling Antibody Sampler Kit (Cell Signaling, CAT 8579).

### Quantitative real-time polymerase chain reaction (qRT-PCR)

RNAiso Plus (Invitrogen, Carlsbad, CA, USA) was used to extract total RNA. Reverse transcription was performed using the RevertAid first-strand cDNA Synthesis Kit (Thermo, Waltham, MA, USA) according to the manufacturer's instructions. qRT-PCR was performed by a SYBR Green Mix kit (Bio Rad Laboratories, Hercules, CA). Analysis of the relative expression was based on the 2^-ΔΔCt^ method. The primers were as follows: GAPDH-forward: 5'-GGTGAAGGTCGGTGTGAACG-3' and GAPDH-reverse: 5'-CTCGCTCCTGGAAGATGGTG-3'; ATP11A-forward: 5'-TACCCAGACAACAGGATCGTC-3' and ATP11A-reverse: 5'-AGCCGTCACAGTAATGACAAAG-3'; MMP2-forward: 5'-CACAGGAGG AGAAGGCTGTG-3' and MMP2-reverse: 5'-GAGCTTGGGAAAGCCAGGAT-3'; MMP9-forward: 5'-TTCAGGGAGACGCCCATTTC-3' and MMP9-reverse: 5'-TGTAGAGTCTCTCGCTGGGG-3'.

### Cell transfection

The plasmids sh-EGFP, sh-ATP11A, Vector and Flag-ATP11A were purchased from Future Biotherapeutics (Suzhou, Jiangsu, China). The sequence was confirmed through DNA sequencing. Lipofectamine 2000 reagent (Invitrogen, Carlsbad, CA, USA) was used for cell transfection based on the manufacturer's protocols. Each well of the 6-well plate contained 2 μg plasmids and 10 μL Lipofectamine 2000.

### Cell migration and invasion assay

Transwell assays were performed using a Transwell inserter (Corning, New York, NY, USA) containing an 8-mm permeability well according to the manufacturer's protocol. Transfected gastric cancer cells were harvested, resuspended in serum-free medium, and transferred to 8 μm permeable wells (5×10^4^ cells/100 µl) while complete medium was added to the chamber bottom. After 20 h, the cells on the upper surface were scraped off, and migrating cells on the lower surface were fixed with 4% polyformaldehyde (Aladdin, Shanghai, China) and stained with 0.05% crystal violet (Beyotime, Shanghai, China) for 30 min. An inverted light microscope (Olympus Corporation) was used to capture the images. The cell invasion assay was similar to the migration assay except that cells were seeded in Matrigel-coated Transwell inserts (BD Bioscience, Corning, NY, USA).

### Wound Healing Assay

25×10^4^ cells/well were seeded in 24-well plates for 24 h and then scratched in wells with a 10 μl micropipette tip. Unattached cells were washed with phosphate-buffered saline (PBS) and fresh medium was added. Wound images were obtained under a microscope at 0 h and 20 h. The wound-healing rate was calculated as follows: 100% × [(wound width at 0 h - width at 20 h)/width at 0 h].

### Cell Counting Kit (CCK)-8 assay

Cell counting kit-8 (CCK-8, Beyotime Institute of Biotechnology, Shanghai) was used to detect cell proliferation. After 48 h of transfection, 1×10^3^ cells/100 μL were seeded into 96-well plates in each well. Then, the cells were incubated with 100 μl CCK8 reagent mixture (10 μl CCK8 reagent: 90 μl DMEM) without light at 37°C for 2 h. The absorbance value was measured at 450 nm by a microplate reader (Bio-Rad, Hercules, CA, USA) and analyzed via GraphPad Prism version 8.

### Colony formation assay

1×10^3^ cells were seeded into each well of 6-well plates and maintained at 37 °C and 5% CO_2_ for 10~14 days. The supernatant was changed every three days. Finally, 4% paraformaldehyde was used to fix and 0.5% crystal violet was used for staining for 30 min. The number of visible colonies was counted by ImageJ.

### Statistical analysis

Data presentation adopted mean ± standard deviation from at least three independent experiments. The t-test and one-way analysis of variance were employed for comparisons between two and multiple groups, respectively. Kaplan-Meier survival analysis was conducted using the log-rank test. The results were analyzed via GraphPad Prism 8 software. *P* < 0.05 was considered statistically significant.

## Results

### ATP11A is aberrantly expressed in gastric cancer tissues

**Figure 1:**
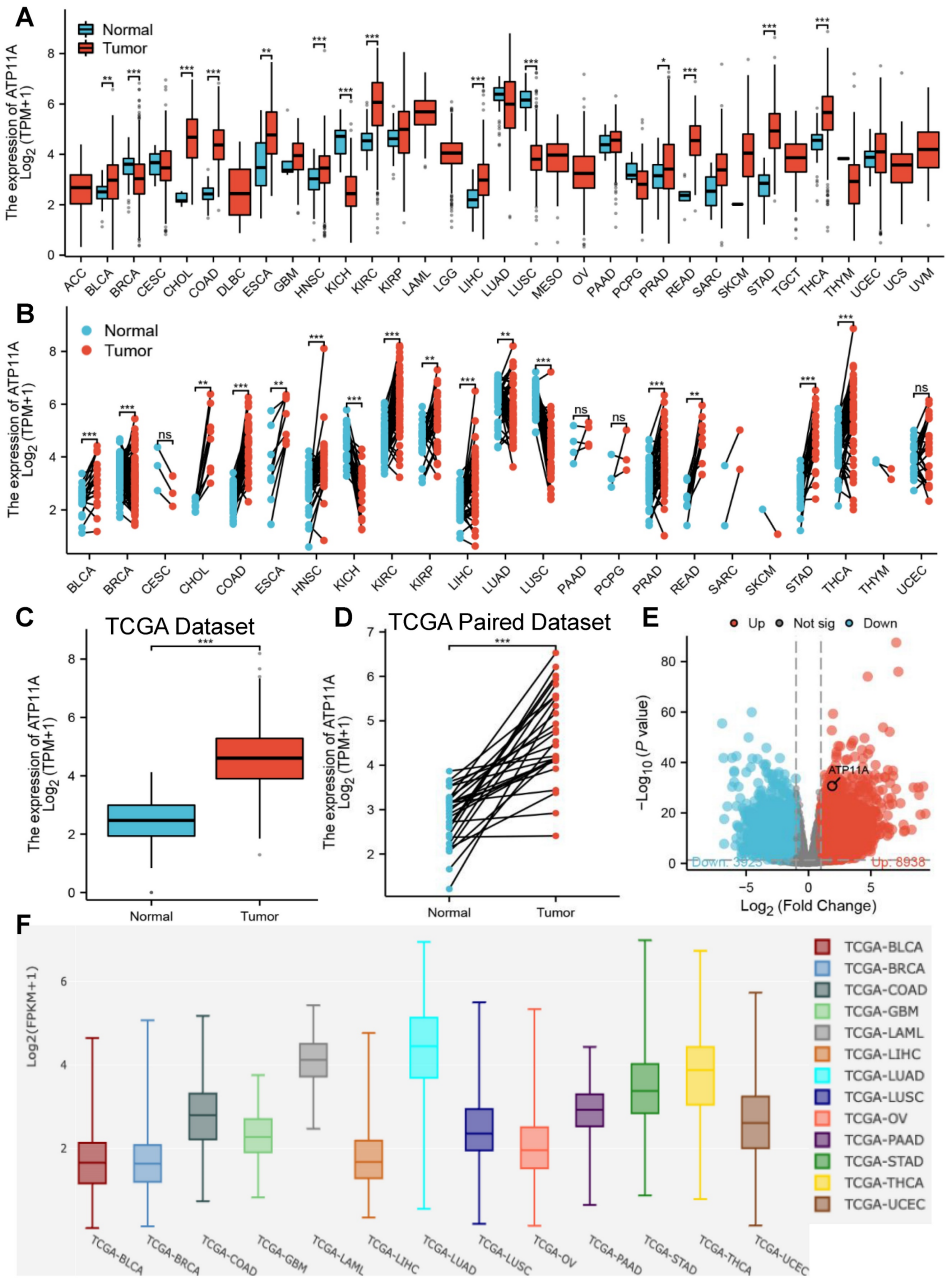
The relative expression of ATP11A in pan-cancer analysis and gastric cancer. (A, B) Different types of tumors compared with normal tissues in TCGA and GTEx databases. (C, D) ATP11A expression in TCGA gastric cancer and normal tissues. (E) The volcano plot of ATP11A expression in TCGA database. (F) The expression of ATP11A in cancer tissue based on single cell database.

The relative expression of ATP11A in cancer tissues and adjacent tissues were explored. Pan-cancer analysis showed that ATP11A was high expression in most of tumors cancer tissues and adjacent tissues including stomach adenocarcinoma (STAD, belong to gastric cancer) in both TCGA paired and unpaired dataset (Figure 1A, B), and the results were verified by in Figure 1C-E. Further comparison of ATP11A expression between tumors in single cell public database (Cancer Single‑cell Expression Map) showed that ATP11A was highly expressed in STAD, Thyroid carcinoma (THCA) and Lung adenocarcinoma (LUAD) amongst other tumors (Figure 1F). These results indicated that ATP11A may serve as an oncogene in gastric cancer.

### ATP11A is associated with poor prognosis in gastric cancer

**Figure 2:**
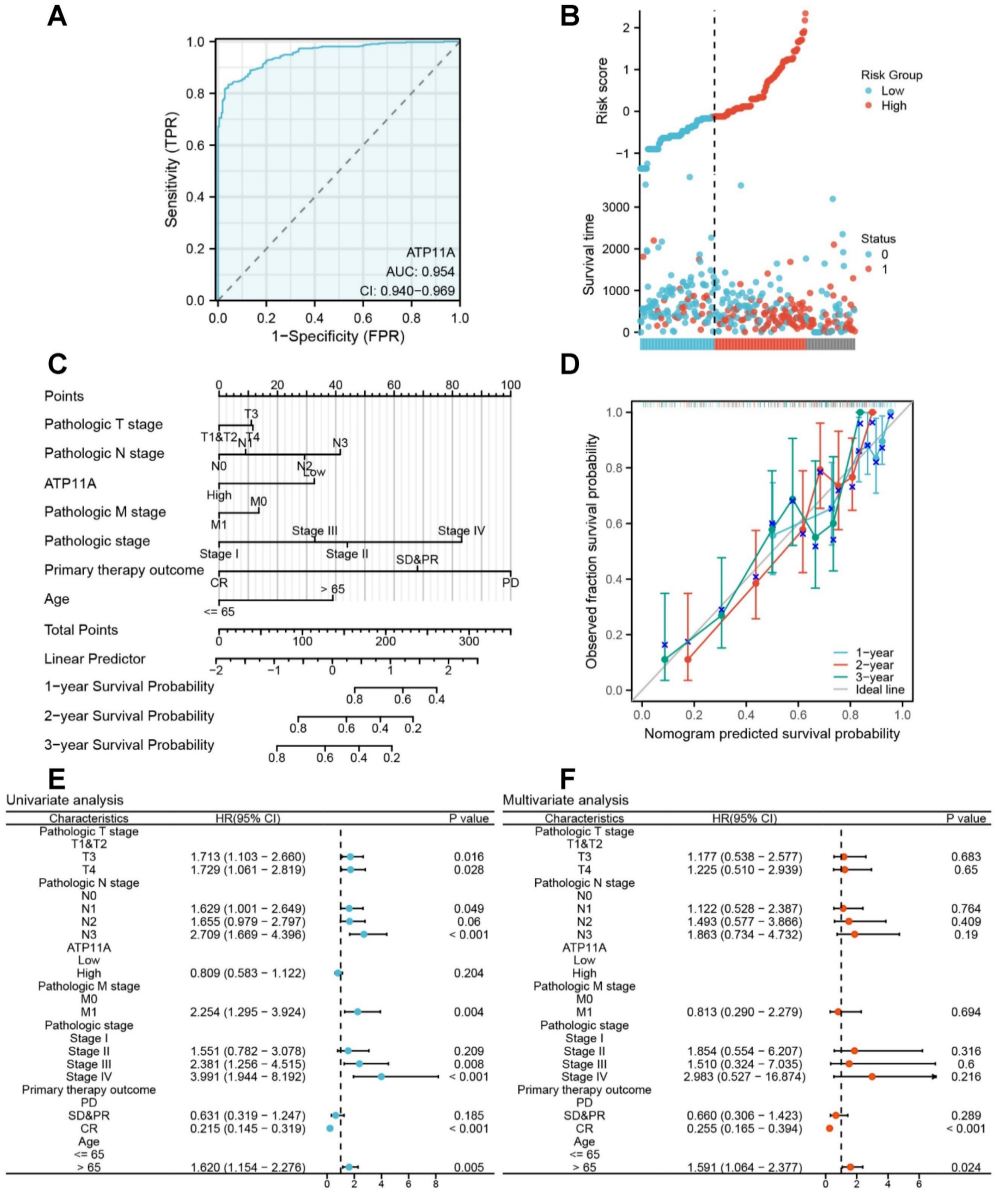
Clinical indicators of ATP11A in gastric cancer. (A) The ROC curve of ATP11A in gastric cancer. (B) The risk score of ATP11A in gastric cancer. (C, D) Nomogram and calibration curves for prediction of 1-, 2-, and 3-year overall survival rates of gastric cancer patients with high ATP11A expression. (E) Forest plot of univariate Cox regression analysis. (F) Forest plot of multivariate Cox regression analysis.

We further explored the effect of ATP11A on the prognosis and clinical significance of gastric cancer. The ROC curve showed that ATP11A had excellent predictive ability to distinguish gastric cancer, with an area under the curve of 0.954 (95% CI=0.940-0.969) (Figure 2A). Risk factor plots further confirmed that ATP11A high expression was associated with poor prognosis (Figure 2B). We further used calibration analysis to predict the association of ATP11A expression with 1, 2, and 3-year prognosis in gastric cancer patients. Meanwhile, we used ATP11A as one of the independent overall survival (OS) factors to construct a prognostic calibration curve for predicting the prognosis of colon cancer patients, and the prediction results suggested that the fit was good, and the survival rate was consistent with the prediction results of the model (Figure 2C, D). Then, the effect of ATP11A on prognosis was analyzed by univariate and multivariate Cox regression analysis. In the univariate analysis, patients with higher expression of ATP11A showed OS in stage T3 (HR: 1.713; Cl: 1.103-2.66; P = 0.016), T4 (HR: 1.729; Cl: 1.061-2.819; P = 0.028), N1 (HR: 1.629; Cl: 1.001-2.649; P = 0.049), N3 (HR: 2.709; Cl: 1.669-4.396; P < 0.001), M1 (HR: 2.254; Cl: 1.295-3.924; P = 0.004), pathological Stage III (HR: 2.381; Cl: 1.256-4.515; P = 0.008), Stage IV (HR: 3.991; Cl: 1.944-8.192; P < 0.001), primary therapy outcome: CR (HR: 0.215; Cl: 0.145-0.319; P < 0.001), Age > 65 (HR: 1.62; Cl: 1.154-2.276; P = 0.005) (Figure 2E). For multivariate analysis, CR (HR: 0.255; Cl: 0.165-0.394; P < 0.001), Age > 65 (HR: 1.591; Cl: 1.064-2.377; P = 0.024) (Figure 2F). The above results suggested that ATP11A can be a potential prognostic factor.

### Related differentially expressed genes (DEGs) and functional enrichment was analyzed in gastric cancer

**Figure 3:**
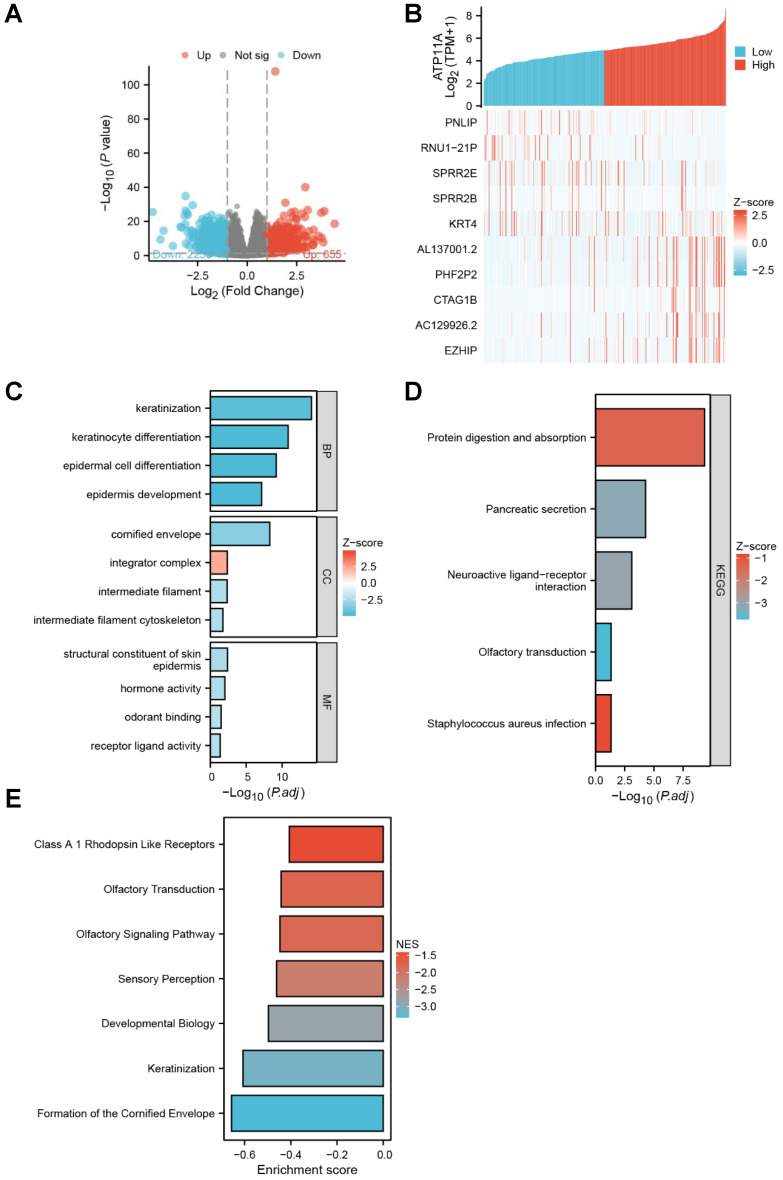
DEGs and Function enrichment of ATP11A in gastric cancer. (A, B) The volcano plot with logFc>1.5 and top 10 differentially expressed genes related to ATP11A from TCGA database. (C-E) KEGG, GO and GSEA analysis of ATP11A and their co-expression genes.

The DSEq2 R package was used to analysis the DEGs of ATP11A in gastric cancer, and the Figure 3A showed that there were 623 differentially expressed genes between the ATP11A high-expression group and the ATP11A low-expression group, including 229 up-regulated genes and 394 down-regulated genes (P < 0.05, |Log2 - FC| > 1.5). Then the relationship between top 10 DEGs (including five top low expression genes as PNLIP, RUN1-21P, SPRR2E, SPRR2B, KRT4 and five top high expression genes as AL137001.2, PHF2P2, CTAG1B, AC129926.2, EZHIP) and ATP11A was shown in Figure 3B. In Go enrichment, ATP11A mainly enriched in keratinization, keratinocyte differentiation, epidermal cell differentiation, epidermis development, cornified envelope, integrator complex, intermediate filament, intermediate filament cytoskeleton, structural constituent of skin epidermis, hormone activity, odorant binding receptor ligand activity (Figure 3C). For KEGG, ATP11A mainly enriched in Protein digestion and absorption, Pancreatic secretion, Neuroactive ligand-receptor interaction, Olfactory transduction and Staphylococcus aureus infection (Figure 3D). For GSEA, ATP11A mainly enriched in Class A 1 Rhodopsin Like Receptors, Olfactory Transduction, Olfactory Signaling Pathway, Sensory Perception, Developmental Biology, Keratinization and Formation of the Cornified Envelope (Figure 3E).

### ATP11A was profiled and promoted migration in gastric cancer cells

**Figure 4:**
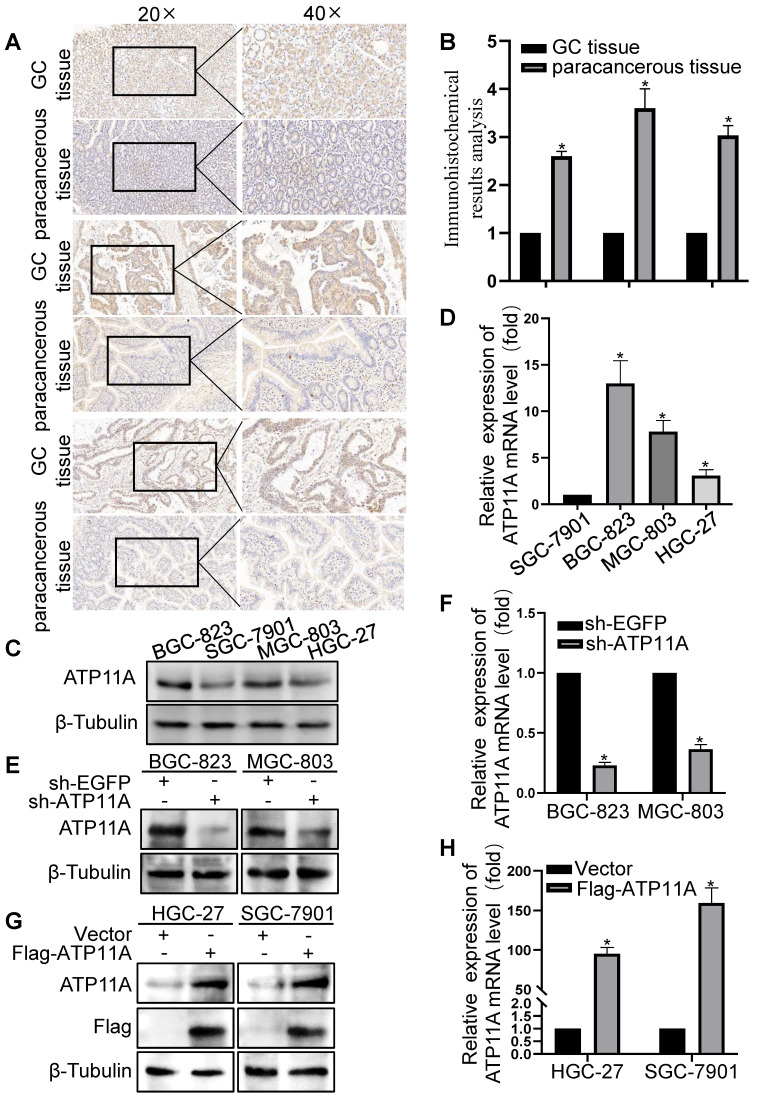
The expression of ATP11A in gastric cancer tissues and cells. (A) IHC staining for ATP11A in gastric cancer and matched paracancerous tissues from three representative patients. Original magnifications 20× and 40× (inset panels). (B) The result analysis of Figure 4A. (C, D) The relative expression levels of ATP11A in different gastric cancer cell lines (BGC-1, SGC-7901, MGC-803 and HGC-27). (E-H) Plasmid transfection efficiency of ATP11A in gastric cancer cells. (*P < 0.05).

Next, we validated the results of bioinformatics analysis in clinical samples and gastric cancer cells. The IHC results showed that ATP11A was upregulated in gastric cancer tissues than in adjacent gastric cancer tissues (Figure 4A-B), and the expression of ATP11A in gastric cancer cells from high to low is BGC-823, MGC-803, HGC-27 and SGC-7901 cells (Figure 4C, D).

**Figure 5:**
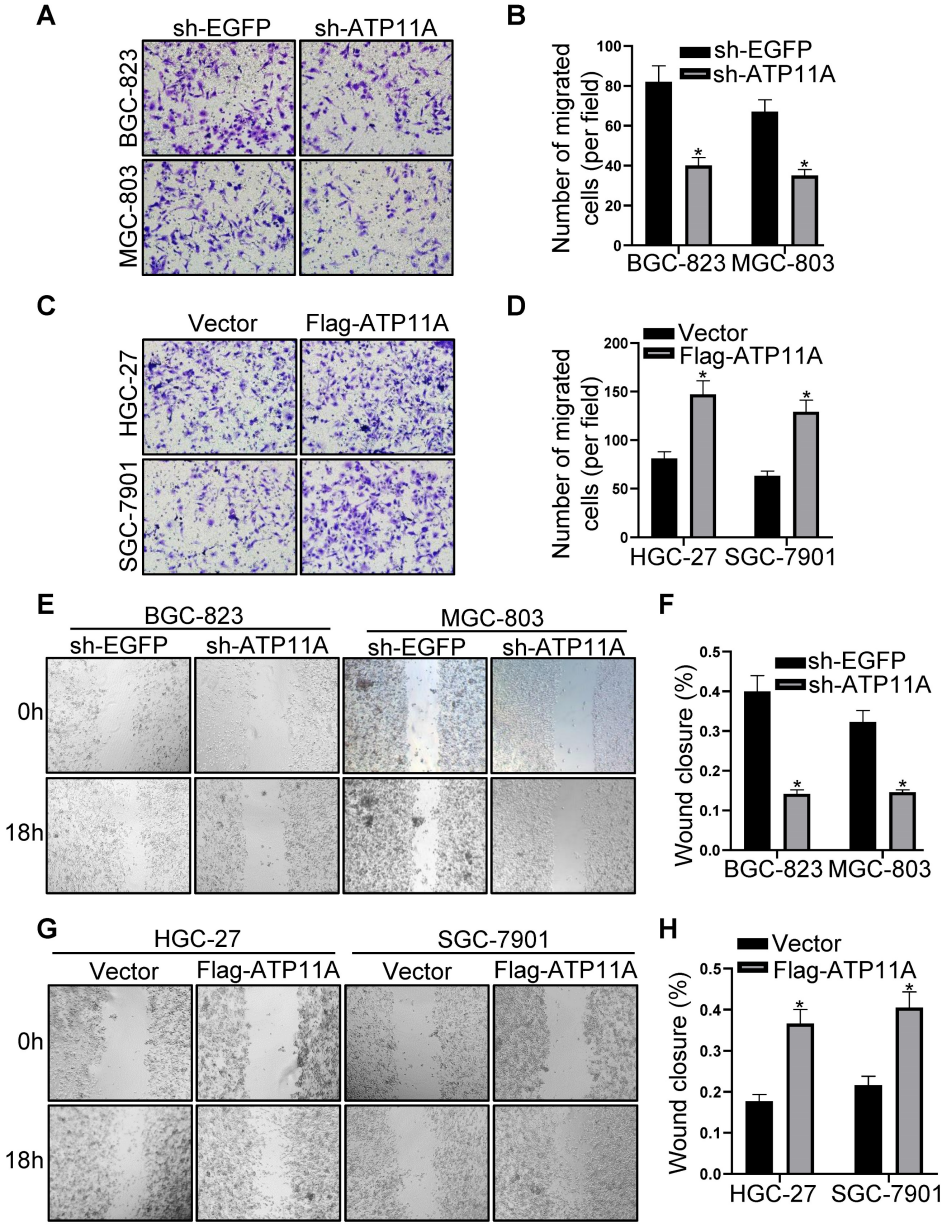
ATP11A promoted migration of gastric cancer cells. (A, B) Transwell assays were used to examine the migration ability of BGC-823 and MGC-803 cells transfected with sh-EGFP and sh-ATP11A. (C, D) Transwell assays were used to detect the migration ability of HGC-27 and SGC-7901 cells transfected with Vector and Flag-ATP11A. (E, F) Wound healing assay showed the migration ability of BGC-823 and MGC-803 cells transfected with sh-EGFP and sh-ATP11A. (G, H) Wound healing assay showed the migration ability of HGC-27 and SGC-7901 cells transfected with Vector and Flag-ATP11A. (*P < 0.05).

Plasmid sh-ATP11A was transfected into BGC-823 and MGC-803 cells with relatively high ATP11A expression while Flag-ATP11A in HGC-27 and SGC-7901 cells with relatively low ATP11A expression, and the results confirmed the efficiency of plasmids (Figure 4E-H). Then, the effect of ATP11A on migration was detected by transwell experiment in gastric cancer cells. The results demonstrated that sh-ATP11A plasmid reduced the number of migrated cells in BGC-823 and MGC-803 cells (Figure 5A, B), whereas many more migrated cells were observed in the Flag-ATP11A group of HGC-27 and SGC-7901 cells (Figure 5C, D). Additionally, ATP11A downregulation weakened the migratory capacity of BGC-823 and MGC-803 cells via the wound healing assay (Figure 5E, F), and ATP11A upregulation enhanced the motility of HGC-27 and SGC-7901 cells (Figure 5G, H). The above results showed that ATP11A promoted the migration of gastric cancer cells.

### ATP11A enhanced the invasion ability of gastric cancer cells

**Figure 6:**
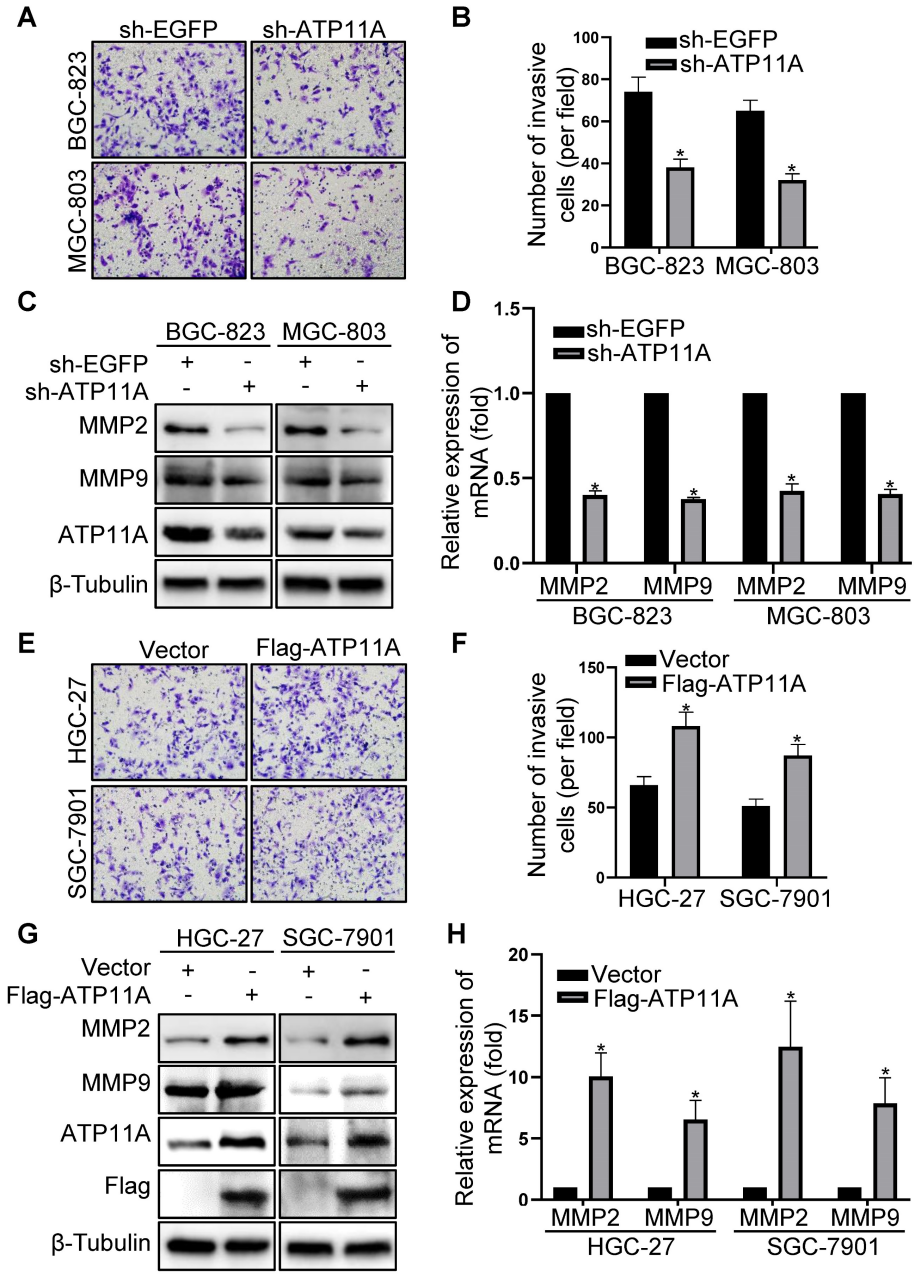
ATP11A enhanced the invasion ability of gastric cancer cells. (A, B) The invasion ability of BGC-823 and MGC-803 cells transfected with sh-EGFP and sh-ATP11A was detected by transwell invasion assays. (C, D) MMP2 and MMP9 expression were examined in BGC-823 and MGC-803 cells transfected with sh-EGFP and sh-ATP11A. (E, F) The invasion ability of HGC-27 and SGC-7901 cells transfected with Vector and Flag-ATP11A was detected by transwell invasion assays. (G, H) MMP2 and MMP9 expression were tested in HGC-27 and SGC-7901 cells transfected with Vector and Flag-ATP11A. (*P < 0.05).

Through additional experiments, we discovered that ATP11A can affect the invasive ability of gastric cancer cells. Compared to the control group, the invasive capability of BGC-823 and MGC-803 cells with ATP11A knockdown was significantly decreased by a Transwell BD assay (Figure 6A, B). Conversely, overexpression of ATP11A in HGC-27 and SGC-7901 cells greatly enhanced invasive ability (Figure 6E, F). In addition, western blotting and qRT-PCR were performed to confirm the role of ATP11A in gastric cancer cell invasion. At both the protein and mRNA levels, MMP2 and MMP9 expression levels significantly decreased in BGC-823 and MGC-803 cells with ATP11A knockdown (Figure 6C, D), while overexpression of ATP11A in HGC-27 and SGC-7901 cells led to a significant increase in MMP2 and MMP9 expression compared to the control group (Figure 6G, H). The above results indicated that ATP11A enhanced the invasive ability of gastric cancer cells.

### ATP11A stimulated gastric cancer cell proliferation

**Figure 7:**
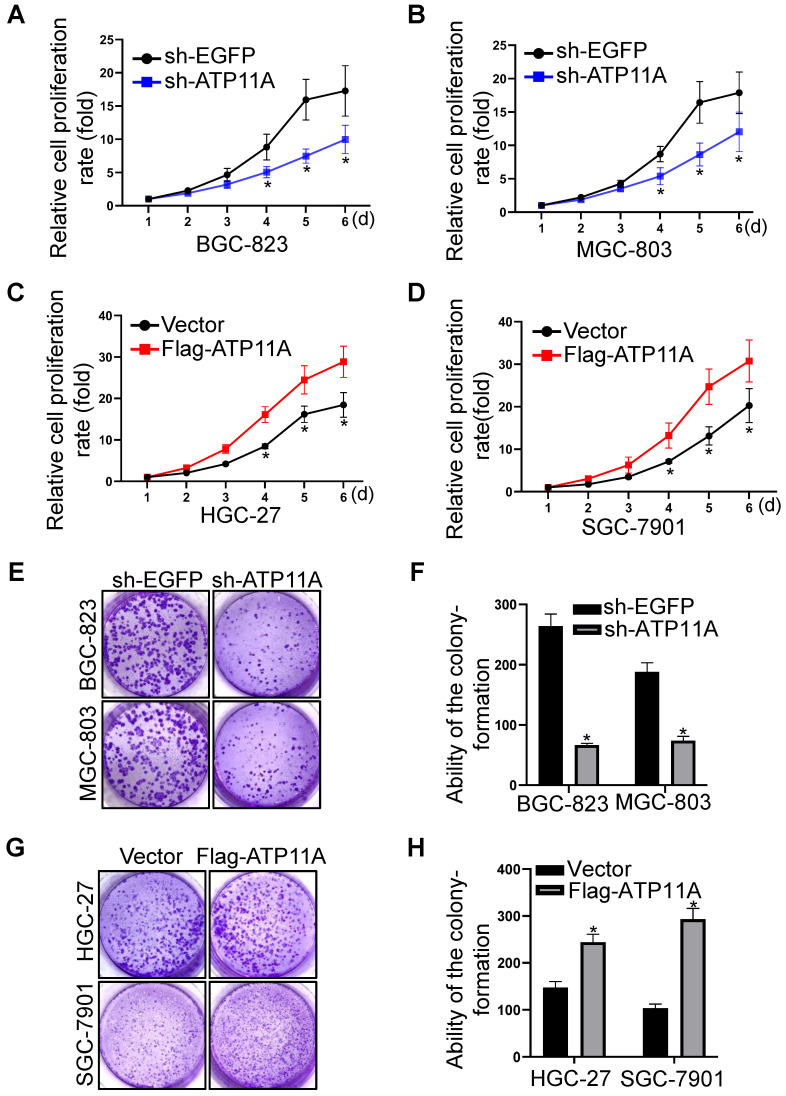
ATP11A promoted the proliferation of gastric cancer cells. (A, B) ATP11A downregulation slowed down the proliferation rate of BGC-823 and MGC-803 cells. (C, D) ATP11A upregulation promoted the proliferation of HGC-27 and SGC-7901 cells. (E, F) ATP11A downregulation weakened colony formation of BGC-823 and MGC-803 cells. (G, H) ATP11A upregulation enhanced colony formation of HGC-27 and SGC-7901 cells. (*P < 0.05).

Furthermore, we explored the role of ATP11A on the proliferation of gastric cancer cells through CCK-8 and colony formation assays. CCK-8 assay showed that compared with the control group, the proliferation ability of BGC-823 and MGC-803 cells with ATP11A knockdown was significantly decreased (Figure 7A, B). Conversely, overexpression of ATP11A in HGC-27 and SGC-7901 cells significantly increased their proliferation abilities (Figure 7C, D). In addition, the colony-forming capacity of BGC-823 and MGC-803 cells transfected with sh-ATP11A was decreased (Figure 7E, F) while ATP11A overexpression enhanced the colony-forming ability of HGC-27 and SGC-7901 cells (Figure 7G, H). These results indicated that ATP11A promoted the proliferation of gastric cancer cells.

### ATP11A induced EMT by devitalizing Hippo pathway in gastric cancer cells

**Figure 8:**
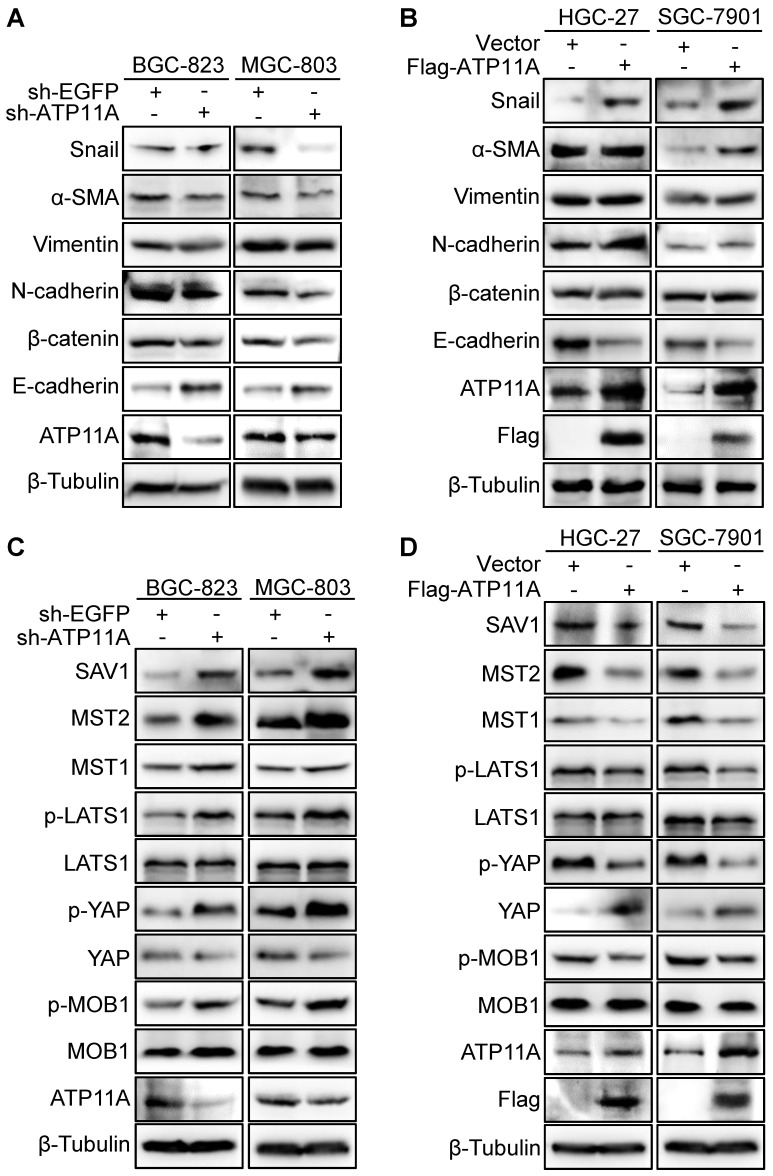
ATP11A promoted EMT in gastric cancer cells via Hippo pathway. (A) Western blot was to examine the expression of EMT proteins in sh-ATP11A-BGC-823 and MGC-803 cells, and (B) Flag-ATP11A-HGC-27 and SGC-7901 cells. (C) The hippo pathway proteins expression was used to detect in BGC-823 and MGC-803 cells with sh-EGFP or sh-ATP11A transfection, and (D) HGC-27 and SGC-7901 cells with Vector or Flag-ATP11A transfection.

Enhanced invasion, migration, and proliferation abilities are usually accompanied by the occurrence of EMT 19. Therefore, the western blot was used to examined EMT markers expression. The results showed that ATP11A knockdown resulted in E-cadherin upregulation and downregulation of N-cadherin, β-catenin, Vimentin, α-SMA and Snail (Figure 8A). Meanwhile, ATP11A upregulation contributed to a decrease in E-cadherin and an increase in N-cadherin, β-catenin, Vimentin, α-SMA and Snail (Figure 8B). As the Hippo pathway plays a vital role in regulating multiple biological processes such as cell proliferation, malignant transformation and cell growth 20, we studied the relationship between ATP11A and the Hippo pathway. Compared to the control group, BGC-823 and MGC-803 cells with ATP11A knockdown exhibited a significant increase in SAV1, MST2, MST1, p-LATS1, p-YAP and p-MOB1 expression, while YAP expression decreased. On the other hand, ATP11A overexpression in HGC-27 and SGC-7901 cells showed a significant decrease in SAV1, MST2, MST1, p-LATS1, p-YAP and p-MOB1 expression, with a increase in YAP expression (Figure 8C, D). In summary, the results indicated that ATP11A can devitalize Hippo signaling to promote EMT in gastric cancer cells.

## Discussion

The present study indicated that ATP11A expression was higher in gastric cancer tissues than normal tissues, and bioinformatic analysis showed that the high expression of ATP11A was related to poor prognosis in patients with gastric cancer. The results of gene enrichment experiments showed that ATP11A was related to epidermal development. Subsequently, we proved that ATP11A led to the increased migration, invasion and proliferation abilities of gastric cancer cells. It was further verified that ATP11A promoted EMT of gastric cancer cells by inhibiting Hippo pathway.

High metastasis and mortality rate and a low 5-year survival rate are the main characteristics of gastric cancer 21. And metastasis has been fully recognized to be related to the EMT of cancer cells 22.

EMT, as a complex reversible process, can be initiated and promoted by cells in the tumor microenvironment and uncontrolled oncogenic signaling pathways, resulting in loss of epithelial cell polarity and intercellular adhesion to participate in the metastasis and invasion of multiple cancers including gastric cancer 23-26. For instance, CST1 regulated GPX4 protein stability to promote gastric cancer metastasis and EMT and inhibit ferroptosis 27. Wu et al also found that CAFLCs-derived fibroblast activation protein facilitated the proliferation, migration, invasion, and EMT of gastric cells 28. In addition, EMT is considered to be a key target for new drugs development to inhibit the progression of gastric cancer 29. Hence, it is necessary to understand the underlying molecular mechanism of EMT in gastric cancer.

The membrane-associated enzyme ATP11A is responsible for lipid flipping, playing a crucial role in maintaining cellular activity by preserving lipid asymmetry at the plasma membrane 30. ATP11A has been reported to be involved in different biological processes of tumor cells. For example, high ATP11A expression was an independent predictor of colorectal cancer 31. Liu et al proved that miR-103a promoted tumor growth and glucose metabolism via ATP11A and EIF5 in hepatocellular carcinoma 32. In pancreatic cancer cells, ATP11A regulated EMT via Numb PRR 18. However, the relationship between ATP11A and EMT in gastric cancer remains unclear. In this study, we first analyzed the expression of ATP11A in TCGA and GTEx databases, and found that ATP11A was overexpressed in gastric cancer tissues compared with normal tissues. Then, we proved that ATP11A is associated with poor prognosis of gastric cancer. It is suggested that ATP11A may play an oncogene role in gastric cancer. Subsequently, we demonstrated that ATP11A promoted the migration, invasion and proliferation of gastric cancer cells. Additionally, we provided evidence that ATP11A could induce EMT in gastric cancer cells. Nevertheless, However, the mechanism by which ATP11A regulates EMT remains unclear.

Aberrant activation of the Hippo pathway signaling is implicated in tumor progression through its promotion of cellular proliferation, development of chemotherapy resistance, and facilitation of metastasis 33. When the Hippo pathway is activated, the activation of MST1/2 leads to a phosphorylation kinase cascade that ultimately results in the phosphorylation of YAP 34. This phosphorylation event subsequently sequesters YAP within the cytoplasm, facilitating its degradation process, nuclear YAP/TAZ promotes cell proliferation and survival signaling 34. Several studies have shown that Hippo signaling pathway plays an important role in EMT of gastric cancer 35-38. Therefore, we hypothesized that ATP11A might promote EMT in gastric cancer cells through Hippo pathway. As expected, we proved that ATP11A significantly inactivated the Hippo pathway to decreased phosphorylation of YAP proteins. Hence, we proposed that ATP11A facilitated the malignant progression of gastric cancer cells via the Hippo/YAP pathway.

To the best of our knowledge, our study is the first to explore the clinical significance and molecular function of ATP11A in gastric cancer. However, there are still some limitations in our research. First of all, the sample collection of gastric cancer patients in our hospital was relatively small, and no western blot and qRT-PCR experiments were performed on tissue samples. Secondly, we overexpressed and knocked down ATP11A in different gastric cancer cell lines, and no animal experiments were performed to verify the cytological results. These problems need to be solved in future research.

In summary, our study showed that the expression of ATP11A in gastric cancer tissues was higher than that in normal tissues. ATP11A was correlated with poor prognosis of gastric cancer. The data from the present study proved that ATP11A promoted the migration, invasion, proliferation and EMT of gastric cancer cells. Furthermore, we demonstrated that ATP11A promoted EMT by inactivating the Hippo pathway in gastric cancer cells. Our study revealed that ATP11A may act as a key role in gastric cancer progression.

## Abbreviations

ATP11A: ATPase phospholipid transporting 11A
EMT: Epithelial-mesenchymal transition
GTEx: Genotype Tissue Expression Project
TCGA: The Cancer Genome Atlas
IHC: Immunohistochemistry
cDNAs: Complementary DNAs
OS: overall survival
PBS: phosphate-buffered saline
SD: standard deviation
ANOVA: analysis of variance
STAD: stomach adenocarcinoma
